# Liquid biopsy-guided kidney-sparing management in upper tract urothelial carcinoma: from preoperative risk stratification to perioperative surveillance

**DOI:** 10.3389/fonc.2026.1844668

**Published:** 2026-06-02

**Authors:** Difei Yu, Jiarun Tang, Yu Zhang, Ke Hu, Jing Qing, Jiamo Zhang

**Affiliations:** Department of Urology, The Affiliated Yongchuan Hospital of Chongqing Medical University, Chongqing, Yongchuan, China

**Keywords:** circulating tumor DNA, kidney-sparing surgery, liquid biopsy, preoperative risk stratification, upper tract urothelial carcinoma, urine biomarkers

## Abstract

Upper tract urothelial carcinoma (UTUC) presents a clinically important gap in urologic oncology: treatment intensity is determined before surgery, yet conventional preoperative risk stratification remains imperfect. Kidney-sparing surgery (KSS) is established for selected low-risk disease and is increasingly considered when renal preservation is clinically important, but safe selection depends on distinguishing technically manageable tumors from biologically unsuitable disease. Computed tomography urography (CTU), cytology, ureteroscopy, and ureteroscopic biopsy remain indispensable, although they describe anatomy and morphology more reliably than tumor biology. Urine-based liquid biopsy platforms, including DNA methylation, mutational, multiplex RNA, copy-number, and protein assays, appear most mature for noninvasive detection and preoperative triage. Plasma circulating tumor DNA (ctDNA), in contrast, appears more closely linked to biological upstaging, occult muscle-invasive or non-organ-confined disease, perioperative risk refinement, and molecular residual disease surveillance. This review follows the KSS decision chain from patient selection to postoperative monitoring. We emphasize that current evidence supports liquid biopsy as an adjunctive, decision-enhancing layer rather than a replacement for imaging, ureteroscopy, pathology, or multidisciplinary judgment. Future studies should move beyond isolated sensitivity and specificity estimates and test whether biomarker-informed pathways improve treatment allocation, renal preservation, surveillance burden, and oncologic outcomes.

## 1 Introduction

UTUC accounts for only 5-10% of urothelial cancers, but its clinical impact is disproportionate (1–3). Compared with bladder cancer, UTUC is more often diagnosed at an invasive stage, is anatomically harder to sample, and frequently requires treatment decisions that can irreversibly alter renal function (2–4). Contemporary epidemiologic series show that presentation and survival patterns have changed over time because of improved imaging, stage migration, and broader recognition of ureteral disease (5–7). This combination of rarity, biological heterogeneity, and treatment consequence makes preoperative decision-making in UTUC especially vulnerable to misclassification. The key clinical problem is not simply diagnosing UTUC, but matching treatment intensity to tumor biology while preserving renal function when this can be done safely. Radical nephroureterectomy (RNU) remains the reference treatment for most high-risk nonmetastatic UTUC, whereas KSS is preferred for appropriately selected low-risk tumors (8–10). Contemporary kidney-sparing options now include endoscopic ablation, segmental ureterectomy, topical chemoablation, and focal therapies, which makes accurate selection more consequential (11–14). In practice, however, this distinction is based on a composite assessment of CTU, cytology, endoscopic appearance, biopsy grade, focality, tumor burden, location, hydronephrosis, renal function, frailty, and patient preference. No single variable is sufficient to determine whether a lesion is genuinely suitable for kidney preservation. This misclassification risk operates in both directions. Overtreatment may remove a renal unit from older patients or from those with chronic kidney disease, potentially limiting future cisplatin eligibility (11, 15, 16). Undertreatment may delay definitive management of occult invasive or biologically aggressive disease and increase the burden of surveillance. The practical question is therefore not how to save kidneys whenever possible, but how to identify patients in whom kidney preservation does not compromise oncologic judgment. It is here where liquid biopsy has taken center stage. Biological reasoning is appealing. UTUC grows in a fluid-exposed urothelial compartment, such that tumor-derived DNA, RNA, epigenetic material, and proteins or cellular surrogates could be identified in urine, and to a smaller but progressively significant level, in blood (17–19). Unlike tissue biopsy, liquid biopsy can be reiterated, longitudinally sampled, and analyzed in a temporal context rather than being a snapshot of an anatomic compartment. It is especially important in UTUC, where standard tissue acquisition is frequently constrained by size, accessibility and depth of sampling (20, 21).

Nevertheless, the discipline must be put into perspective. The usefulness of liquid biopsy will be determined by the ability to address clinically significant problems. In the case of UTUC and KSS, these issues are particular. Does a urine test decrease uncertainty preceding ureteroscopy or repeat ureteroscopic observation? Is it beneficial in separating between lesions which are just detectable and those, which are biologically worrisome? Is plasma ctDNA able to distinguish the patient who already has a disease that acts more like muscle-invasive cancer or non-organ-confined cancer in spite of the unclear local results? And after conservative treatment, can serial urinary or plasma tumor DNA detect early failure in a way that meaningfully improves surveillance? Those are the questions that now define whether liquid biopsy will become clinically relevant in UTUC.

Recent literature suggests that this is no longer a purely theoretical discussion. Urine-based assays have matured rapidly over the past few years, and prospective or validation studies across methylation, mutation, RNA, copy-number, and protein platforms are summarized in **Table 1** (18, 22). Plasma ctDNA remains less mature in localized UTUC, but the available evidence is more directly tied to biological upstaging, poor prognosis, and perioperative risk refinement (23–25). In parallel, broader urothelial cancer literature has shown that molecular residual disease detection can carry treatment and surveillance implications (26–28).

**Table 1:** Key urine-based liquid biopsy studies for diagnosis and preoperative triage.

Study	Biomarker/assay	Setting	Key finding	Potential relevance to KSS	Main limitation
Freund et al., 2018 (51)	UroVysion FISH	Selective upper-tract urine; surveillance/triage	Sensitivity 90%, specificity 80% in a feasibility setting.	Could reduce unnecessary follow-up URS after KSS.	Very small cohort.
Hayashi et al., 2019 (60)	TERT/FGFR3 urinary cfDNA	Preoperative diagnosis and stage enrichment	FGFR3 signal associated with ≤pT1 disease; combined assay improved diagnostic performance.	Suggests biologically informative urine profiling beyond cytology.	Panel limited; exploratory stage signal.
Xu et al., 2021 (61)	17-gene mutation panel + ONECUT2 methylation	Preoperative detection	High diagnostic accuracy in suspected UTUC.	May improve triage before invasive work-up.	Internal validation only.
Territo et al., 2022 (55)	Urine methylation panel	Prospective diagnostic setting	Outperformed cytology, especially for high-grade disease.	Useful adjunct when false-negative cytology is a concern.	Single-centre.
Ouyang et al., 2022 (62)	Point mutation + methylation model	Hematuria/diagnostic triage	Promising discrimination in multicentre framework.	Supports integrated molecular triage before URS.	Diagnosis-focused, not outcome-guiding.
Ghoreifi et al., 2023 (49)	DNA methylation marker test	Prospective cohort	High sensitivity and specificity for UTUC detection.	Clinically relevant adjunct to preoperative triage.	Direct KSS outcome data unavailable.
Palermo et al., 2024 (57)	Bladder EpiCheck	Selective upper-tract urine	Particularly strong signal for high-grade disease.	May enrich for lesions less suitable for conservative management.	Limited outcome linkage.
Wei et al., 2024 (58)	Urine liquid biopsy, self-matched design	Preoperative detection	Markedly higher sensitivity than cytology.	Reduces false reassurance before KSS decisions.	Small cohort.
Zhang et al., 2024 (22)	8-gene RNA panel	Prospective multicentre validation	Robust noninvasive discrimination before treatment.	One of the strongest current pre-URS triage data sets.	Still not a standalone KSS selector.
Yang et al., 2026 (50)	UroCAD copy-number assay	Prospective multicentre study	High diagnostic performance; CNV burden may correlate with aggressiveness.	Potentially valuable for risk enrichment beyond simple detection.	Needs broader external and decisional validation.
Fujita et al., 2026 (63)	Oncuria-Detect multiplex immunoassay	Noninvasive detection	High sensitivity/NPV profile.	Likely more useful for suspicion triage than definitive selection.	Specificity more modest than sensitivity.

KSS, kidney-sparing surgery; RNU, radical nephroureterectomy; URS, ureteroscopy; CTU, computed tomography urography; ctDNA, circulating tumor DNA; cfDNA, cell-free DNA; MRD, molecular residual disease; NOC, non–organ-confined; CNV, copy-number variation.

## 2 Current clinical boundaries of kidney-sparing surgery in UTUC

Any discussion of liquid biopsy in UTUC should begin with the clinical boundary conditions of KSS. Across major guideline systems, the principle is stable: low-risk localized UTUC is the main domain of kidney preservation, whereas RNU remains the reference standard for high-risk nonmetastatic disease (8–10, 29). The details are different, and nevertheless, the general arrangement is not. Still, guidelines are based on the synergies of radiologic, cytologic, endoscopic and pathologic features to detect favorable disease, and realize that the same features are not always concordant (8, 9, 30).

This model has evolved to be more subtle. Initial epidemiologic and preoperative assessments reviews highlighted the relative infrequency of UTUC and the paucity of evidence on the disease (3, 4). Recent reviews have moved the discourse to the comparative effectiveness, long-term preservation of renal, surveillance burden, and development of nonextirpative modalities (11, 15, 31, 32). This development is significant in the sense that it transforms the meaning of “appropriate treatment”. Whether a conservative approach can, or can ever, be oncologically acceptable, is no longer the central question. The more pertinent question is what patients are supposed to be given which method of conservatism and of what degree of biological confidence.

KSS is not a single procedure. It can be defined as a family of organ preserving strategies, which have varying suppositions of the location, burden, accessibility, and local control of a tumor. Endoscopic ablation is the most common and common modality especially where the lesions are of low grade with good anatomy. The least invasive procedure of organ preservation is ureteroscopic management; although, it also relies on the quality of endoscopic staging, grading, and visualization the most (11, 32). The segmental ureterectomy takes another niche. It is more particularly applied to distal or mid-ureteral tumors that can be resected using endoscopic renal function-preserving reconstruction and provide a more definitive local specimen and a better tradeoff between endoscopic surveillance and oncologic control in some patients (1, 12).

There is an increasing systematic evidence in favor of KSS. The systematic review by Seisen of the EAU is still fundamental in indicating that favorable tumors may yield the same oncologic results as those of RNU and be conservatively treated (33). Rather than reversing that picture, more contemporary reviews have modernized it. Head and Raman pointed out that the practical argument no longer revolves around the question of whether KSS “works” should be used in low-risk disease, but rather about what one should prefer between ablation, segmental surgery, chemoablation, and emerging focal therapies (11). Schuil and colleagues emphasized the increasing body of evidence on KSS effectiveness in a broader range of indications although they also emphasize the fact that much evidence base is still retrospective (31). Another similarity between Ko and others was that kidney-sparing management has been presented as an emerging, yet promising field, motivated by more accurate diagnostic tools and new therapeutic agents, not by any dramatic individual randomized data set (32).

The therapeutic environment has been expanded as well. The OLYMPUS trial made UGN-101 a topic into the mainstream dialogue as a kidney-sparing agent to treat select low-grade upper tract disease (13, 34). WST-11, when used as vascular-targeted photodynamic therapy has been another useful focal therapy with promising early outcomes in patients with residual or recurring disease following a previous endoscopic intervention or in patients who could not undergo surgery (14). Surveys of intraluminal and endocavitary therapy also indicate that the space spared to the kidney is no longer limited to mechanical ablation only (15, 35). These innovations have not however eliminated the fundamental issue. They have amplified it. The greater the amount of alternatives that can be used to preserve the renal unit, the better it becomes to know what types of tumors can indeed be used in terms of conservative management.

One of the primary causes of this change is renal functioning. RNU continues to be reminded with perioperative reviews that extirpative surgery has not only oncologic but also renal and systemic morbidity (16). This becomes particularly crucial in UTUC since a significant number of patients are elderly, have baseline chronic kidney disease or are also prone to ineligibility to receive platinum-based systemic therapy post-nephroureterectomy (11, 16). In this sense, kidney preservation cannot be regarded as a quality- of -life issue. It is a more comprehensive issue of treatment-sequencing. In cases where kidney saving is important, the price of excess treatment is elevated.

Meanwhile, management that is conservative comes at a price. Old and modern research studies on intravesical recurrence following RNU indicate that UTUC management is not limited to the local treatment but the behavior of the tumor is dynamic beyond that of the surgery (36, 37). In the case of KSS, the effect of this dynamic is increased. Conservative treatment subjects the patients to more frequent monitoring, periodic endoscopy, and the prospect of delayed radical surgery. Therefore, the “benefit” of KSS would never be termed as the preservation of the kidney at any cost. The advantage is that the kidney is saved without the loss of the adequate oncologic control.

This balance is even less stable when clinicians get to classic low-risk disease. A number of recent reviews describe the growing application of KSS in the selected settings beyond the strictest definition of low-risk groups, particularly favorable ureteral tumors or in the patient with imperative indications (15, 31, 32). This extension is clinically explicable. Still, it also reveals the frailty of selection which is all morphology. The more distant the field is to the intuitive low-risk disease the greater the reliance of the field on superior biologic discrimination.

The contemporary KSS landscape should then be cautiously avoided to be caricatured as a plain two column guideline table. In reality, the process of making decisions is stratified. It integrates the tumor characteristics, technical feasibility, patient priority, age, comorbidity, the history of the patient with bladder cancer, and his proximity to close follow-up. These are practical considerations which justify why the same lesion can be treated differently in two patients. They also specify the reason as to why an extra biological indicator, provided reliable enough, may want to meaningfully modify management even without substituting any traditional examination.

KSS is thus seen as a management approach rather than a process. Its safety is reliant on a closer correspondence between tumor biology and amount of treatment than is traditionally needed under extirpative surgery. This is the very reason why the level of preoperative and perioperative stratification has such significance in UTUC. **Table 2** demonstrates that, even kidney-preserving decisions are still mostly dependent on composite risk estimation as opposed to individual preoperative variable. That being the case, the necessity of a more biologically informative layer is self-evident.

**Table 2:** Current clinical boundaries of kidney-sparing surgery in UTUC.

Domain	Current position	Why it matters for KSS	Representative refs
Risk framework	Integrated clinicopathologic stratification; no molecular biomarker is validated for routine use yet.	Supports the manuscript premise that KSS depends on composite risk estimation rather than a single test.	(8–10)
Low-risk disease	KSS is guideline-supported when findings are concordantly favorable and close surveillance is feasible.	Defines the clearest evidence-based space for organ-preserving management.	(8, 9, 33)
High-risk disease	RNU remains the reference treatment for most high-risk nonmetastatic UTUC.	Frames the threshold that liquid biopsy must challenge only cautiously.	(8–10)
Imperative indications	Renal preservation may be prioritized in solitary kidney, bilateral disease, or chronic kidney disease.	Explains why better biologic triage matters even more when nephron loss has systemic consequences.	(11, 15, 16)
Segmental ureterectomy	A kidney-sparing option particularly relevant for selected ureteral tumours with favorable anatomy.	Illustrates that KSS is a strategy with multiple modalities, not endoscopy alone.	(11, 12, 32)
Chemoablation/focal therapy	UGN-101 and focal ablative approaches expand the KSS toolbox in selected low-grade disease.	Increases the need for accurate patient selection before conservative treatment.	(13, 14, 34)
Surveillance burden	KSS trades extirpation for a longer and more intensive follow-up pathway.	Strengthens the rationale for biomarkers that can refine both upfront selection and later surveillance.	(15, 31, 37)

KSS, kidney-sparing surgery; RNU, radical nephroureterectomy; URS, ureteroscopy; CTU, computed tomography urography; ctDNA, circulating tumor DNA; cfDNA, cell-free DNA; MRD, molecular residual disease; NOC, non–organ-confined; CNV, copy-number variation.

## 3 Why conventional preoperative stratification is still not enough

Liquid biopsy will only gain clinical significance when the constraints of the standard pathway are outlined in an honest manner. The work-up of UTUC still depends on CTU, cytology, ureteroscopy, and ureteroscopic biopsy (30). They are not incidental historical artifacts. They have become the norm of these days as they present a set of information that cannot be substituted by any biomarker: anatomic localization, visual confirmation, tissue diagnosis, and procedural planning. The problem is not that these tools fail in a global sense. The problem is that each tool leaves a biologically important zone of uncertainty, and that uncertainty matters most when the clinician is deciding whether a kidney can be safely preserved.

CTU remains the imaging backbone of diagnosis and local staging. Reviews of diagnosis and risk stratification continue to place CTU at the center of the initial work-up because it identifies filling defects, obstruction, hydronephrosis, local anatomy, and the overall extent of upper tract involvement (4). That role is unlikely to change soon. However, CTU is better at showing where disease is than at proving how aggressive that disease is. Suspicious radiologic features can increase concern for invasive behavior, but they do not reliably distinguish all lesions that are technically manageable from those that are biologically unsafe for conservative treatment. This limitation is especially relevant in the gray zone of apparently low-volume or localized disease.

Urinary cytology has almost the opposite problem. A clearly positive cytology result—especially in the high-grade setting—can be highly informative, but sensitivity is variable and often disappointing, particularly for low-grade lesions (38–40). The 2016 meta-analysis of selective upper tract cytology by Potretzke and colleagues confirmed that specificity was favorable but sensitivity remained only moderate (38). Later studies of urinary cytology in UTUC and upper urinary tract cytology by pre and post-implementation of The Paris System were similar: cytology is helpful but it is not a reliable sole-gatekeeper to treatment decisions (39, 40). In the case of KSS where false reassurance is especially expensive, this constraint has practical implications.

Diagnostic ureteroscopy was expected to solve some of these problems by allowing direct visualization and biopsy. It has certainly improved local assessment and remains indispensable when imaging and cytology are inconclusive or when tissue confirmation will change management (41, 42). Yet ureteroscopy is not a perfect biological arbiter. It is invasive, it may need to be repeated, and the biopsy material obtained is often small and superficial. In many patients, particularly those being considered for endoscopic treatment, the clinician is trying to infer the overall biology of the lesion from a limited sample collected through a narrow anatomic window.

This concern is well supported by the literature. Simon and colleagues showed the imperfect concordance between upper urinary tract cytology, biopsy, and final surgical pathology in a large cohort (43). Subiela and colleagues then quantified the problem in a systematic review and meta-analysis, confirming that ureteroscopic biopsy has meaningful limitations in predicting final stage and grade (20). These findings have not been challenged by more recent discourses; on the contrary, they have increased the clinical significance of these findings in the kidney-sparing era. It is an undergraduation and understaging which has been well summarized by Jue and colleagues as the comment is not a primary evidence paper; however, one of the strongest strengths of KSS is the factor of inaccurate histopathologic diagnosis which has been pointed out as one of its greatest weaknesses (44).

Other series support the same argument. The discordance between ureteroscopic biopsy and final pathology was specifically studied by Margolin and colleagues and it was revealed that the mismatch occurs not as an oft-repeated event (21). Surveys on the position of ureteroscopy in the contemporary diagnosis have also determined that even prudent endoscopic examination do not completely close the disparity amid what is examined and what the tumor is really (41, 42). It explains the capabilities of biopsy and its inabilities.

The other underappreciated limitation that is not fully appreciated is that the traditional route treats the uncertainty of preoperative and postoperative as two different problems. As a matter of fact they are interrelated. Under the circumstances of low preoperative confidence, the burden of postoperative surveillance increases. That is why KSS may easily dictate regular ureteroscopy and strict follow-up despite the presence of initially favorable lesions in the patients. The diagnostic uncertainty prior to the treatment is effectively passed over to the more vigorous follow up schedule of treatment. A biologically informative biomarker need not be useful because it did not substitute a single preoperative test because it could result in less uncertainty that had to be addressed using the invasive surveillance.

This can be said of intravesical recurrence and downstream management as well. The predictors of the recurrence following RNU have been reported over the years and the less invasive series published recently indicate that the patterns of recurrence are not irrelevant in the postoperative management of UTUC (36, 37). It unfolds over time. A single tissue diagnosis is relevant, but not all dynamic biological processes which determine recurrence and progression. This is time-dependent, which is one of the reasons why liquid biopsy is conceptually appealing: it can be done as a longitudinal change and not as a single baseline event that should be interpreted.

And more fundamentally there is the problem. In the present risk model, morphology and clinicopathologic surrogates continue to be used as the primary building blocks. However, genomic and molecular research that has been done in the last ten years has revealed UTUC is not bladder cancer in another place (45–47). Molecular classification studies conducted by Fujii. revealed that UTUC is a disease that is susceptible to being classified into biologically distinct groups with significant diagnostic and treatment implications (47). Sfakianos and others have also reviewed studies pointing to higher expression levels of FGFR3-associated changes and other lineage-related variations as important genomic differences between upper tract and bladder urothelial carcinoma (45, 48). Biology hence concerns UTUC, yet the majority of actual preoperative choices are still made with the help of tools that partially address that biology.

**Figure 1:**
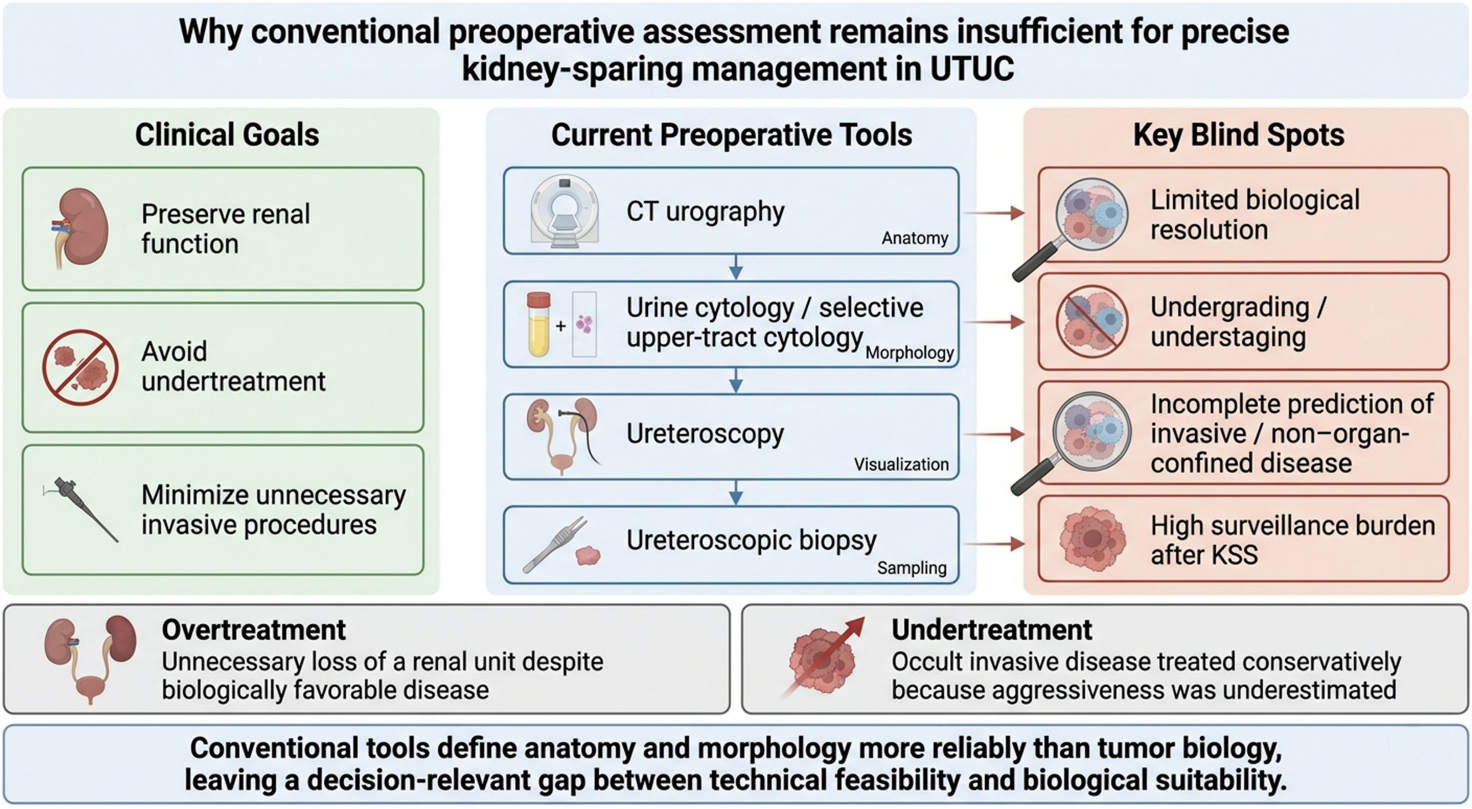
Why conventional preoperative assessment remains insufficient for precise kidney-sparing management in UTUC. The figure aligns the clinical goals of renal preservation with the current preoperative tools and the blind spots that remain when morphology-based evaluation is used to select KSS.

This is why **Figure 1** models the incompatibility of the clinical objective of renal preservation and the existing preoperative tools as well as the blind spots that precede the choice of treatment. **Table 3** maps each conventional tool to its major strengths and to the specific limitations that drive undergrading, understaging, and transfer of uncertainty into surveillance. The need for liquid biopsy does not arise because clinicians want more tests. It arises because current tools leave a decision-relevant blind spot between technical feasibility and biologic suitability. If a biomarker cannot narrow that gap, then it is unlikely to change KSS in a meaningful way. If it can, then even an adjunctive role could be important. The next section therefore focuses on the most developed part of the field: urine-based liquid biopsy for preoperative triage and patient selection.

**Table 3:** Strengths and limitations of conventional preoperative evaluation tools.

Tool	Primary role	Major strengths	Key limitations relevant to KSS	Representative refs
CT urography	Initial diagnosis and staging	High diagnostic accuracy; defines anatomy and hydronephrosis.	May miss subtle/flat disease and incompletely reflect biologic aggressiveness before KSS.	(3, 4, 30)
Voided/selective cytology	Noninvasive adjunct for grade-oriented assessment	High specificity, especially for high-grade disease.	Moderate sensitivity; false reassurance remains a problem in low-grade or limited disease.	(38–40)
Diagnostic ureteroscopy	Direct visual assessment and targeted sampling	Clarifies anatomy and allows lesion-directed evaluation.	Invasive; repeat procedures add burden and visual appearance still imperfectly reflects biology.	(41, 42)
Ureteroscopic biopsy	Tissue confirmation and approximate grading	Essential when management depends on histology.	Sampling error, undergrading, and understaging remain important.	(20, 21)
Clinicopathologic models	Integration of imaging, cytology, grade, size, and focality	Useful real-world framework for triage.	Still built mainly on morphology and surrogates rather than direct tumor biology.	(8, 30, 75)

KSS, kidney-sparing surgery; RNU, radical nephroureterectomy; URS, ureteroscopy; CTU, computed tomography urography; ctDNA, circulating tumor DNA; cfDNA, cell-free DNA; MRD, molecular residual disease; NOC, non–organ-confined; CNV, copy-number variation.

## 4 Urine-based liquid biopsy for preoperative patient selection

**Figure 2:**
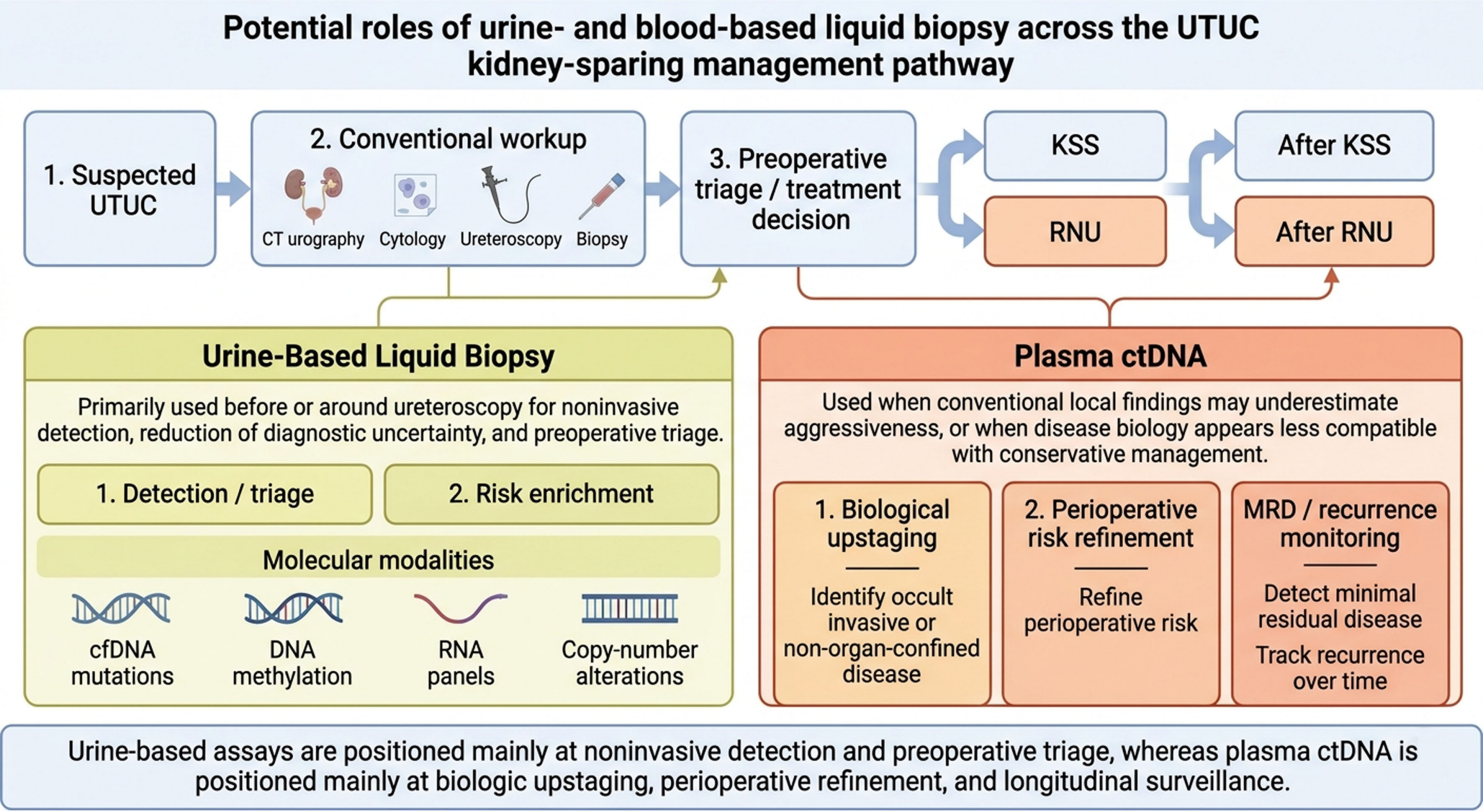
Potential roles of urine- and blood-based liquid biopsy across the UTUC kidney-sparing management pathway. Urine-based assays are positioned primarily at the diagnostic and preoperative triage stage, whereas plasma ctDNA is shown as a tool for biologic upstaging, perioperative risk refinement, and longitudinal surveillance.

Urine is the most intuitive fluid compartment for biomarker development in UTUC. The tumor is directly bathed by urine, sample acquisition is simple, repeatable, and inexpensive, and tumor-derived material can in principle be captured without tissue removal. For these reasons, urine-based liquid biopsy has advanced more rapidly in localized UTUC than blood-based assays for several practical applications (17). The strongest current evidence supports urine-based testing for noninvasive detection and preoperative triage. Whether these tests can safely influence KSS selection more directly is the more difficult question, and the answer is still evolving. The intended clinical division of labor between urine assays (front-end detection and triage) and plasma ctDNA (biologic upstaging and perioperative risk refinement) is outlined in **Figure 2**.

Not all urine-based assays are clinically informative in the same way. Some primarily strengthen noninvasive detection, whereas others are more relevant to preoperative triage or to identifying lesions that may be biologically less suitable for conservative treatment. This difference is an issue in UTUC. In kidney-sparing decision-making, sensitivity in diagnosis is insufficient and whether a biomarker produces some meaningful decrease in uncertainty regarding tumor aggressiveness and treatment appropriateness is the more critical question. In that regard, clinical usefulness of the urine-based liquid biopsy is not so much about ascertaining the presence of a lesion than making it clear that it seems to be safe to preserve it in renal.

### 4.1 Detection and triage: moving beyond cytology

This is one point that the recent urine biomarker literature puts across in a very clear manner: cytology is no longer the sole realistic noninvasive comparator. Recent validation studies of methylation, RNA, and copy-number platforms, together with earlier fluorescence *in situ* hybridization (FISH)-based studies, have broadened this comparison beyond conventional cytology (22, 49–53). This systematic review by Białek. demonstrated the extent to which the noninvasive biomarker space had already diversified by 2022, although the challenges of translation still remain as candidate-proven and limited by small samples (54). More recent publications have gone beyond proof-of-concept and into prospective or multicenter validation, especially on methylation, RNA, and copy-number platforms. (**Table 1**) (22, 49, 50).

This change is important since the practical question is not always the mere question whether a test is positive. In most of the patients suspected to have UTUC, the more relevant data is to determine whether the negative result is reliable enough to lessen the immediate uncertainty. A high-sensitivity urine assay with good negative predictive value may help in at least two scenarios. The first is the patient with suspicious symptoms or imaging but nondiagnostic cytology. The second is the patient already on a kidney-sparing pathway who faces repeated ureteroscopic surveillance. In both settings, the most immediate value of liquid biopsy may be to reduce false reassurance from a weak or negative cytology result.

The older FISH literature helps illustrate both the promise and the limits of this approach. Freund demonstrated that the technology of UroVysion FISH on 1 mL of selectively collected urine was technically practical and promising sensitivity in a small feasibility study with the problem of follow-up as a central theme following KSS (51). The study was small, but its clinical framing was important: the goal was not biomarker novelty for its own sake, but outpatient triage to reduce the need for repeated ureteroscopy. Su and colleagues also found that voided urine FISH positivity correlated with invasive and high-grade disease, which was also argued to indicate that FISH could have more than a binary diagnostic message (52). This was subsequently demonstrated by Sassa. that an improved diagnostic rate is obtained with the combination of UroVysion and traditional cytology in the detection of upper tract urothelial carcinoma, especially in the high-grade environment (53). These figures do not render FISH an ultimate choice of treatment, but they depict a primary tenet that still drives the discipline: the most beneficial urine tests are useful since they alter the quality of preoperative doubt.

Triage-oriented profile of other urine platforms has demonstrated a similar pattern, with sensitivity and negative predictive value that might be most helpful when the task at hand is reduction of pre-endoscopic uncertainty as opposed to treatment definition alone (54).

### 4.2 DNA methylation assays: the most mature urine-based platform

Methylation testing is now one of the most developed and clinically appealing platforms of urine-based methods. These are both biological and practical reasons. The alterations of the DNA methylation are stable and can be measured in the urine even in the case of the limited cell yield, and connected to the premature tumor-related changes. A number of recent reports now lend credence to the idea that, in some cases, methylation testing can offer better diagnostic capabilities than traditional urine testing, and that, on the whole, the body of evidence tends to concur on the same (49, 55, 56).

The most recent study that is also the strongest one in the prospective cohort is that by Ghoreifi and colleagues. Their urine-based DNA methylation marker of urine test had good sensitivity and good specificity in the detection of UTUC (49). Not only the headline numbers are important in this study. The reason is that the assay was assessed in a clinically significant preoperative setting and obviously compares better to what clinicians typically are relying on as a result of routine cytology. Such an outcome is especially applicable in cases where the danger of undertreatment is more important than the inconvenience of further testing.

The article by Territo and colleagues contributes to the complementary view. In their single-center prospective study, urine methylation biomarkers showed useful performance characteristics in the diagnosis of UTUC, and the authors explicitly positioned the assay as an aid to a pathway in which current tools do not always allow correct disease staging and grading (55). Palermo and colleagues subsequently evaluated Bladder EpiCheck prospectively in upper urinary tract urine samples and showed a profile that was especially compelling for high-grade disease (57). This is an important nuance. In the KSS setting, a test that performs particularly well for high-grade lesions may be more valuable than one that performs equally across all biological categories. The main danger in conservative management is not missing every lesion. It is underestimating the lesions that should not be managed conservatively.

Wei and colleagues approached the same problem from a slightly different angle in a self-matched study of urine-based liquid biopsy, again reporting substantially stronger sensitivity than cytology (58). Taken together, these methylation-focused studies support a defensible near-term conclusion: urine methylation testing is ready to be viewed as a useful “adjunct to conventional preoperative triage”. What remains unproven is whether a favorable methylation profile can independently justify KSS in an otherwise equivocal case. That is a higher level of evidence than any current methylation study provides.

Meta-analytic evidence adds weight but also caution. Lin and colleagues reviewed the methylation architecture of UTUC and linked epigenetic alterations to both diagnosis and prognosis, while emphasizing the heterogeneity of available evidence (59). Ye and colleagues then performed a dedicated systematic review and meta-analysis of DNA methylation urine testing and found strong pooled diagnostic performance, but they also highlighted substantial heterogeneity and the need for better-organized validation studies (56). These findings are useful for a review article because they support the strength of the signal without justifying overstatement. Methylation assays appear promising and, in some settings, clinically useful. They are not yet the substitutes of ureteroscopic assessment and pathology.

### 4.3 Mutation-based urinary DNA: toward biology-informed triage

Urinary assays based on mutation have an alternative appeal. Theoretically, they can also give not just a detection signal but also a biologically relevant read out that can be more directly linked to stage, grade or lineage. One of the first important examples was given by Hayashi and others who investigated urinary cell-free DNA to detect TERT promoter and FGFR3 hotspots mutations (60) and found that a combined molecular testing and cytological approach has a better diagnostic sensitivity than a high specificity. Just as important, FGFR3 positivity was seen only in ≤pT1 tumors, suggesting that urinary genomic information may help enrich for lower-stage disease.

This is the kind of observation that matters in a KSS-centered review. It does not prove that urinary FGFR3 or TERT testing can define a safe conservative-treatment population. But it does suggest that noninvasive urine genomics may do more than merely confirm the presence of urothelial carcinoma. It may begin to shift the probability that a lesion belongs to one biological category rather than another.

Xu and colleagues moved this concept forward with a urine-based assay combining a 17-gene mutation panel and ONECUT2 methylation (61). Ouyang and colleagues similarly developed a combined urinary point mutation and methylation approach with encouraging diagnostic results in hematuria-based cohorts (62). These studies are best understood as steps from diagnosis toward biologic triage. Their clinical value does not depend on replacing tissue. Instead, they help refine the question that tissue is being asked to answer.

The most balanced way to interpret the mutation-based literature is therefore probabilistic rather than binary. A favorable urinary molecular profile may increase confidence that the lesion is biologically less aggressive, whereas an adverse or high-burden profile may raise concern that “technically feasible” does not equal “safely preservable.” It is a significant difference. The conservative management decisions are usually made in the state of uncertainty. A biomarker does not have to offer absolute certainty in order to be clinically useful. It just has to reduce the uncertainty towards a direction that alters management or a follow-up.

### 4.4 RNA, copy-number, and multiplex assays: expanding the translational range

RNA-based and copy-number-based amplitude of investigation has enhanced the sphere with superior biological dosage alongside, in other instances, deeper validation structures. The most critical one of the latest examples is the massive prospective validation of an eight-gene urine RNA test in European Urology Oncology (22). This assay demonstrated good discrimination between UTUC and non-UTUC controls in a treatment-naïve setting and had an amount of prospective multicenter validation that was frequently missing in other biomarker studies.

The relevance of the RNA literature is based not only on the metrics of performance but also workflow implications. The eight-gene test specifically targeted a pre-treatment environment where the existing methods are still invasive, expensive, and inefficient with early and low-grade tumors (22). That is particularly applicable to the front end of a KSS pathway. A test which can lessen the use of diagnostic ureteroscopy in patients who are selected or at least rank the patients in order of need of invasive confirmation, deals with a true clinical bottleneck.

Even more interesting in risk enrichment may be the copy-number based testing. Yang and others also mentioned prospective multicentric validation of the UroCAD assay indicating high diagnostic sensitivity to detect UTUC (50). In addition to diagnosis, copy-number variation (CNV) burden could provide data on tumor aggressiveness. In the event that that association is also strong in larger datasets and more clinically-annotated cohorts, copy-number testing might be more applicable than mere detection-oriented markers to KSS decisions.

Even further diversification of the platform is the multiplex protein testing. According to Fujita, Oncuria-Detect multiplex immunoassay was capable of detecting and classifying UTUC and differentiating between patients with cancer and nontumor-bearing individuals (63). This profile seems to be particularly appealing in the case of noninvasive rule-out strategies. Once again, though, it is not the KSS question as to whether the assay is statistically impressive in the broad sense. Whether its performance characteristics are appropriate to a clinically meaningful decision node or not is an issue. In the case of Oncuria-Detect, it appears that the short term opportunity is more in the area of suspicion triage and surveillance than in the area of establishing the definitive treatment eligibility.

### 4.5 What urine-based liquid biopsy can and cannot currently do for KSS

At this stage, the urine biomarker literature confirms three claims, which are reasonably secure. First, there are a number of urine-based assays with better sensitivity than conventional cytology, and some of them still have clinically useful specificity or negative predictive value (22, 49, 56). Second, the current generation of UTUC biomarkers is supported by more validation data than the previous generations because of their methylation-based and RNA-based platforms (55, 56). Third, not all assays can provide complete biologic data: in particular, mutation-based and copy-number-based methods can provide partial data that is not just presence-or-absence data (50, 57, 60).

That being the case, the existing urine biomarker literature is yet to substantiate a number of clinical inferences that are stronger. No existing assay is safe enough to substitute diagnostic ureteroscopy in all patients with suspected UTUC and no urine marker can be able to define low-risk disease in a manner that would eliminate the need to conduct tissue testing and imaging. More to the point, there is still no prospective evidence that biomarker-based preoperative selection is actually enhancing kidney-sparing outcomes, be it in terms of delayed radical surgery, burden of recurrence, or burden of surveillance. That is, there has been an increase in assay performance which is greater than the decisional validation. The biomarkers are getting stronger, and the data required to be able to facilitate the direct choice of treatment remains incomplete.

As it was summarized in **Table 1**, the literature of urine-based liquid biopsy does not stay at the detection level. A large portion of the existing evidence remains in the area of diagnostic performance and preoperative triage, although a smaller proportion of the literature is also indicating biologic risk enrichment. In the case of UTUC, this difference is important since the clinical question does not include the presence of a lesion, but whether its behavior is in line with safe kidney-sparing treatment. Urine-based liquid biopsy in the said scenario is better regarded as a decision-supporting tool rather than an alternative to endoscopy or pathology.

## 5 Plasma and urine tumor DNA across the perioperative pathway

Assays on urine seem to be most applicable at the initial stages of the UTUC pathway, such as noninvasive detection, and during preoperative triage. In comparison, plasma ctDNA and tumor-informed urinary DNA are more pertinent to another group of clinical questions such as biological upstaging, perioperative risk refinement, and postoperative surveillance of residual disease or early failure (64). As summarized in **Table 4**, the extant evidence regarding localized UTUC suggests that the assays might serve as useful tools that help determine tumors that show more aggressive behavior than what conventional work-up would suggest. Within that context, their clinical utility is less screening than uncovering biologic risk which can change intensity and follow-up of treatment.

**Table 4:** Key plasma and urinary tumor DNA studies in perioperative UTUC management.

Study	Analyte/timing	Clinical question	Key finding	KSS/perioperative implication
Hayashi et al., 2019 (60)	Urinary cfDNA (TERT/FGFR3), preoperative	Can urinary tumor DNA enrich for lower-stage disease?	FGFR3 mutation detected only in ≤pT1 tumours; cytology combination improved performance.	Supports biologic staging at the front end of a KSS pathway.
Nakano et al., 2022 (23)	Plasma ctDNA, perioperative	Can ctDNA identify poor-prognosis localized UTUC?	Preoperative ctDNA fraction >2% and postoperative ctDNA positivity were associated with poorer outcomes.	Highlights biological risk not fully captured by conventional work-up.
Huelster et al., 2024 (24)	Plasma ctDNA + copy-number burden, preoperative	Can ctDNA predict muscle-invasive/non–organ-confined disease?	ctDNA and CN burden predicted MI/NOC disease before surgery.	Most direct evidence that plasma ctDNA may challenge conservative-treatment reassurance.
Tamura et al., 2024 (25)	Tumor-informed plasma/urinary ctDNA, postoperative	Can longitudinal ctDNA detect recurrence early?	Serial individualized monitoring detected postoperative recurrence dynamics.	Supports molecularly informed surveillance after KSS or RNU.
Powles et al., 2021/2024 (26, 66)	Postoperative ctDNA in urothelial carcinoma	Does ctDNA identify residual-risk states relevant to adjuvant treatment?	ctDNA positivity marked poorer prognosis and informed benefit signal in adjuvant setting.	Translational support for MRD-oriented escalation in UTUC.

KSS, kidney-sparing surgery; RNU, radical nephroureterectomy; URS, ureteroscopy; CTU, computed tomography urography; ctDNA, circulating tumor DNA; cfDNA, cell-free DNA; MRD, molecular residual disease; NOC, non–organ-confined; CNV, copy-number variation.

### 5.1 Preoperative biological upstaging

In the case of KSS, recurrence is not the most important error. It is choosing a patient to undergo conservative therapy whose pathology was not biologically predisposed to it. Plasma ctDNA seems to be applicable specifically because it can possibly determine that subgroup. In UTUC, Nakano reported one of the first explicit perioperative prognostic indicators in the form of an above 2% ctDNA fraction during the preoperative period (23), and positive ctDNA levels after surgery were also poor prognostic factors. The trial was not intended to be a treatment-allocation trial but showed that ctDNA had an ability to reflect clinically significant biological risk in localized UTUC.

In an European Urology research, this idea was brought to a surgical decision point by Huelster and colleagues, using preoperative plasma ctDNA and copy-number burden to predict muscle-invasive and non-organ-confined UTUC in a more clinically relevant manner than either model alone and pairwise (24). The implication in practice is easy to grasp. Whenever the conventional local evaluation indicates that the lesion could be technically controlled but the ctDNA evaluation is pointing to an increased risk of invasive or extra-organ disease, the practitioner should be more alert to de-escalate treatment. It is not equivalent to claim that ctDNA should be used alone to decide who is given neoadjuvant chemotherapy and who has to go through RNU. It implies that ctDNA has the ability to reveal the flaws of morphology-based reassurance.

### 5.2 ctDNA and treatment intensity across the perioperative continuum

This fact becomes significant even more when KSS is considered in the bigger treatment chain as opposed to a local intervention applied in isolation. UTUC decisions do not just have local control. They also affect the work of the kidneys, the possibility of cisplatin treatment, and the time interval of a perioperative systemic therapy (65) A patient undergoing RNU may lose a renal reserve; a patient undergoing KSS may save their kidney but expose themselves to the risk of late detection of aggressive illness. Plasma ctDNA is at the point of convergence of these opposing realities. It may help identify patients whose disease biology is already leaning toward a more aggressive pathway.

The available evidence does not support using plasma ctDNA as a routine standalone trigger for perioperative systemic therapy in localized UTUC. Current studies remain small, observational, and largely noninterventional (23, 24). A more defensible interpretation is that preoperative plasma ctDNA may help identify patients whose disease biology appears less compatible with conservative management and more consistent with intensified staging, multidisciplinary review, or discussion of radical and perioperative treatment strategies. Even without randomized biomarker-stratified trials, that level of biological risk refinement is already clinically relevant.

The translational support for that position is stronger when the broader urothelial cancer literature is considered. In the IMvigor010 ctDNA analysis, postoperative ctDNA positivity identified a subgroup with poor prognosis that appeared to benefit from adjuvant atezolizumab, whereas ctDNA-negative patients did not show the same treatment advantage (66). Those studies are not UTUC-specific and should not be presented as direct evidence for KSS in upper tract disease. But they do support a broader principle that is highly relevant here: ctDNA does not merely document residual disease. It may define a therapeutically relevant biological state.

The same broader principle is emphasized in recent bladder- and urothelial-focused reviews. Stewart and colleagues framed perioperative ctDNA as an emerging tool for guiding treatment intensity in urothelial carcinoma (27). Bellmunt and colleagues described the likely near-term integration of ctDNA testing into bladder cancer management, particularly for muscle-invasive and metastatic disease (67). For UTUC, these data do not remove the need for disease-specific validation. But they help explain why perioperative ctDNA has moved from an interesting concept to a realistic translational priority.

### 5.3 Postoperative recurrence detection and molecular residual disease

If preoperative biological upstaging is one possible role for ctDNA, postoperative surveillance may be the other major entry point. After treatment, the clinical question changes. The surgeon is no longer trying to decide whether the lesion is amenable to conservative resection. The question arises whether residual disease exists or recurrence is coming up sooner than the conventional surveillance is able to discover. This is in harmony with molecular residual disease logic.

Tamura directly addressed this in a prospective study of individualized plasma and urinary ctDNA following RNU based on the fact that patient-specific tumor-informed monitoring could identify recurrence dynamically over time and included evidence of the viability of both plasma and urinary tracking (25). This is very specifically in the case of UTUC since conventional surveillance is thorough, protracted and not perfected into an individual. Any biomarker that would predict or detect the risk of recurrence early or increase or decrease the timing of monitoring would not just include laboratory information. It would handle one of the overall operational impediments of kidney-sparing and post-nephroureterectomy care.

KSS and RNU slightly differ in terms of their implications. Following KSS, the next highest value of urinary or plasma tumor DNA can be early identification of local failure or intravesical recurrence which would possibly lead to an early ureteroscopic reevaluation or salvage therapy. Following RNU, more emphasis can be put on molecular residual disease (MRD), risk of relapse, or postoperative intensification trial enrollment. The conceptual step forward is identical in both of these environments: surveillance is able to be more biological, and it is no longer entirely tied to strict timetables.

A further limitation is temporal discordance. Plasma ctDNA positivity may precede radiographic or cystoscopic recurrence, but the interval between molecular detection and clinically actionable disease is not yet standardized in localized UTUC. Conversely, a local upper tract recurrence after KSS may shed more readily into urine than into plasma, whereas urinary tumor DNA can be confounded by bladder recurrence, concurrent bladder urothelial carcinoma, field cancerization, inflammation, hematuria, or recent instrumentation. For this reason, molecular positivity should currently prompt earlier clinical reassessment rather than pre-emptive treatment in the absence of anatomical, endoscopic, cytological, or pathological corroboration.

Such distinction is significant as it explains what sort of evidence remains missing. A trial demonstrating that ctDNA is a replacement of imaging is not necessary. That is not the likely future. A more realistic and, more pertinent to UTUC, a pathway where ctDNA changes how and when clinicians take action following treatment is more realistic. Clinically useful but never completely replacing conventional imaging or endoscopy, a biomarker to identify what patients will require increased vigilance, earlier ureteroscopy, or more vigorous discussion of adjuvant therapy may be useful.

### 5.4 The perioperative evidence is promising, but it is not yet decisive

One of the key difficulties in the interpretation of the literature of interpreting the perioperative ctDNA is the inclination to confuse prognostic association with an immediate clinical course of action. At present, that step remains premature. As shown in **Table 4**, the UTUC-specific studies are still limited by relatively small cohorts, heterogeneous assay design, nonuniform sampling time points, and the absence of prospective evidence showing that ctDNA-guided management improves patient outcomes (23–25). In addition, the clinical meaning of a positive perioperative ctDNA signal depends heavily on context. A biomarker associated with recurrence after radical nephroureterectomy does not automatically provide a validated basis for selecting or excluding kidney-sparing treatment before intervention. These questions are related, but they arise at different points along the treatment pathway and should not be conflated. The current evidence instead supports a more selective interpretation. Preoperative plasma ctDNA is most relevant when the concern is occult invasive or non–organ-confined disease, whereas postoperative tumor-informed plasma or urinary testing appears more informative for molecular residual disease and early treatment failure. As reflected in **Table 5**, the current ctDNA data are most useful when interpreted alongside conventional assessment rather than against it. Their principal localized UTUC value does not lie in their ability to substitute imaging, ureteroscopy or pathology, but instead, to contribute biological resolution in cases where the intensity of treatment and surveillance is unclear. This may be especially pertinent where the clinical issue of concern is occult aggressive disease, residual disease or early recurrence as opposed to diagnosis. Plasma and urinary tumor DNA thus are amongst the most promising emerging tools in this setting, although their application is still supportive, not conclusive. Prospective validation at the clinical nodes where administration is likely to change should then be the next move as opposed to general unstructured adoption.

**Table 5:** Proposed integration of liquid biopsy into the UTUC KSS decision chain.

Clinical scenario	Candidate liquid biopsy	What it may add	Potential management impact	Representative refs
Hematuria/suspected UTUC before URS	Voided urine methylation, RNA, mutation, or CNV panels	Increase noninvasive diagnostic confidence and enrich pre-test probability.	May triage which patients need immediate URS versus repeat assessment.	(18, 22, 49)
Equivocal cytology or discordant local work-up	Urine-based liquid biopsy	Reduce false reassurance from negative or weak cytology.	Can support repeat sampling, closer review, or stronger caution before KSS.	(38, 55, 58)
Technically favorable but biologically uncertain lesion	Urine biomarkers and/or plasma ctDNA	Add a biology-informed layer when morphology alone is inconclusive.	May refine KSS candidacy or prompt intensified staging and MDT review.	(24, 60, 62)
Preoperative concern for invasive/NOC disease	Plasma ctDNA	Identify occult aggressive disease despite limited local evidence.	May support discussion of RNU and perioperative systemic therapy when concordant with conventional evidence.	(23, 24)
Post-KSS surveillance	Urinary or plasma tumor-informed DNA	Detect early failure or recurrence before rigid schedule-based surveillance alone.	Could individualize follow-up intensity.	(25, 51)
Post-RNU surveillance	Plasma/urinary ctDNA for MRD	Define residual-risk states and recurrence dynamics.	Supports MRD-oriented surveillance and future escalation trials.	(25, 26, 28)

KSS, kidney-sparing surgery; RNU, radical nephroureterectomy; URS, ureteroscopy; CTU, computed tomography urography; ctDNA, circulating tumor DNA; cfDNA, cell-free DNA; MRD, molecular residual disease; NOC, non–organ-confined; CNV, copy-number variation.

## 6 A liquid biopsy-enhanced framework for KSS decision-making

**Figure 3:**
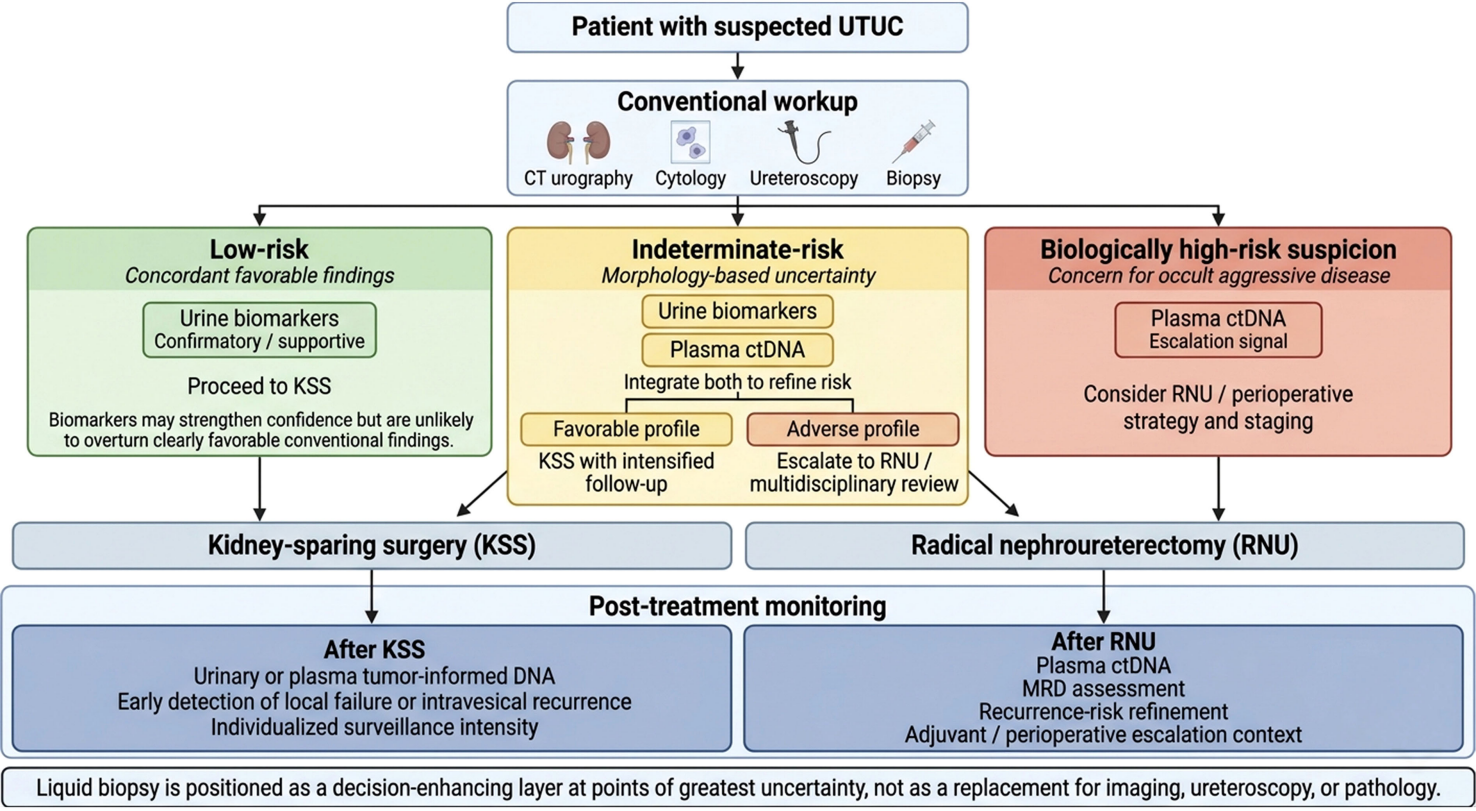
Proposed liquid biopsy-enhanced algorithm for kidney-sparing decision-making in UTUC. The scheme illustrates how urine biomarkers and plasma ctDNA may be inserted at low-risk, indeterminate-risk, and biologically high-risk decision nodes. In indeterminate-risk cases, adverse molecular findings are intended to prompt repeat assessment, intensified staging, MDT review, and closer surveillance rather than automatic conversion to RNU.

In UTUC, the clinical utility of liquid biopsy is unlikely to arise from a single best assay. It is more likely to come from selective use of molecular information at points where conventional assessment leaves clinically important uncertainty. Liquid biopsy should not substitute for imaging, ureteroscopy, or pathology; rather, it should refine risk when conventional findings are discordant or incomplete. As illustrated in **Figure 3**, the framework can be understood across three decision contexts: concordant low-risk disease, indeterminate-risk disease, and biologically high-risk suspicion, with different monitoring implications after KSS versus RNU. This is least apparent in the case of concordant low-risk disease patients. In cases where the imaging, cytology, ureteroscopic appearance, and biopsy have all been supportive of the same favorable interpretation, the urine-based liquid biopsy is unlikely to make a change by itself, and there is no strong reason why it should. Its role in this environment will be more likely confirmatory or longitudinal such as by reinforcing belief in conservative management or by recommending a less intrusive surveillance policy should traditional results appear reassuring but not absolutely conclusive. In this case, the information on the biomarkers should be viewed as a supplement than definitive.

The indeterminate-risk population is the most relevant setting for biomarker integration. This includes patients with localized imaging but equivocal cytology, low-grade biopsy with adverse radiological or endoscopic features, or technically manageable lesions that still raise concern for occult aggressiveness. In this setting, biomarker findings should be reviewed within a multidisciplinary team (MDT) rather than interpreted as isolated triggers for treatment escalation. Urologists, radiologists, pathologists, and medical oncologists should jointly determine whether discordant results warrant repeat sampling, intensified staging, closer surveillance, systemic therapy evaluation, or conversion to radical treatment. For patients with biologically high-risk suspicion, a strongly positive plasma ctDNA result, high-burden adverse urinary molecular profile, or molecular pattern consistent with invasive or non-organ-confined disease should raise the threshold for KSS. However, these signals should not function as automatic switches to RNU. Their most defensible current role is to trigger repeat assessment, intensified staging, MDT review, and closer alignment with conventional evidence. RNU or perioperative systemic therapy should be considered when the molecular signal is concordant with imaging, cytology, ureteroscopic findings, biopsy, or the broader clinical context. Postoperative management is also redefined using the same framework. Liquid biopsy can possibly be most benefitful after KSS, as an early failure detection layer to add to traditional surveillance, especially in cases where the intent is to detect recurrence before it is manifested in a clinical way. Following RNU, focus is put on molecular residual disease, refinement of recurrence-risk and ultimately integrated into perioperative escalation trials. The conceptual framework of both settings remains the same: liquid biopsy should be taken rather as a decision-supporting factor of the clinical process, than the alternative to the established examination.

The given interpretation is still in close correlation with the existing evidence. Biomarkers on urine remain more developed than plasma ctDNA in diagnostic and preoperative triage, and plasma ctDNA seems to be of more application in biological upstaging and perioperative escalation. The most naturally applicable tumor-informed longitudinal methods are applied to surveillance and molecular residual disease. These functions are complementary and not interchangeable and their worth lies in finding the appropriate biomarker to the appropriate stage in the treatment pathway.

## 7 Special scenarios in which better biological stratification may matter even more

Certain clinical scenarios increase the effects of preoperative uncertainty. The most frequent reason against nephroureterectomy is seen in patients with a solitary kidney, bilateral tumors, or advanced chronic kidney disease renal failure. But they also are the patients where undertreatment can be the most harmful. With this kind of environment, even a small biological risk estimation upgrade would have disproportionate practical significance. The argument is not that liquid biopsy has already been proven to be applicable in such situations. It is because such situations would be best to explain why a biology-enhanced decision layer would be important in clinical practice.

Distal ureteral tumors are also worth being treated separately (12). In these patients, segmental ureterectomy may also provide a kidney-saving option but at the same time is more concerned with which kidney-saving modality offers the best balance between local control, preservation of renal function and surveillance burden. Enrichment biomarkers of biologically desirable disease may have an effect on this decision, although may not definitively determine it.

It is another dimension with Lynch syndrome. UTUC is a recognized component of the Lynch syndrome tumor spectrum, and recent reviews have emphasized broader or selective mismatch repair-based molecular assessment in appropriately selected patients, while universal mismatch repair (MMR) screening in UTUC has also been systematically evaluated (68, 69). Wu and colleagues also demonstrated that inherited pathogenic variants can be higher than previously thought in the select populations of UTUC based on the tumor size, grade and location alone (70). Family history and genomic setting could also be of value in prognosis and surveillance requirement especially in those patients where the consequences of diagnostic error have more clinical implications.

Though the existing evidence is not such as to warrant different biomarker algorithms to these subgroups, it does imply that the merit of enhanced preoperative biological stratification perhaps is highest specifically in these high-stakes environments. These are probably some of the most informative populations to test liquid biopsy in case it is to play a significant role in kidney-sparing management.

## 8 Barriers to implementation

Lack of biomarkers is not the greatest obstacle. It is the difference in the performance of biomarkers and their clinical utility. Numerous studies currently indicate that UTUC can be detected by a urine or plasma test or predict recurrence. Much fewer demonstrate that the use of assay alters the practice of clinicians or enhances the experience of patients. The difference is particularly essential in KSS, since the outcome in question is not the diagnostic accuracy as such. It is improved correlation between tumor biology and intensity of treatment.

Assay heterogeneity remains a major barrier. Studies differ in specimen type, collection timing, DNA or RNA input requirements, sequencing depth, methylation cutoffs, mutation-calling strategy, control populations, and clinical endpoints (18, 27, 56, 64). Some enroll hematuric patients, others enroll patients already planned for RNU, others address postoperative prognosis, and some measure only diagnostic discrimination. Sensitivity and specificity estimates from these small, heterogeneous cohorts often have overlapping confidence intervals and should not be pooled informally as if they answer the same clinical question. Specimen-source heterogeneity is equally important. Selectively collected upper tract urine may provide better spatial resolution for a suspected upper tract lesion, but it requires instrumentation and is not equivalent to voided urine. Voided urine is easier to obtain and more scalable, yet it can contain DNA, RNA, methylation signals, or cells derived from the bladder, contralateral upper tract, inflammation, hematuria, or recent manipulation. Concurrent or prior bladder urothelial carcinoma is a particularly important confounder because it can reduce the spatial specificity of urine-based liquid biopsy in patients being evaluated for UTUC.

Evidence from broader urothelial cancer cohorts provides supportive but indirect context for urine-based molecular testing in UTUC. Urinary comprehensive genomic profiling can capture clinically relevant somatic alterations in urothelial carcinoma, but these findings should be extrapolated cautiously to UTUC-specific KSS selection (71). Similarly, studies combining Bladder EpiCheck with cytology and urinary methylation analyses in bladder or mixed urothelial cohorts support complementarity with cytology and recurrence-risk stratification, but they do not by themselves validate a molecular trigger for kidney-sparing eligibility or conversion to RNU in UTUC (72, 73). Broader reviews of immune-related markers such as programmed death-ligand 1 (PD-L1) in advanced bladder cancer further illustrate urothelial molecular heterogeneity, although they should be regarded as background evidence rather than direct evidence for localized UTUC KSS decisions (74).

False-positive and over-escalation risks must also be considered. Molecular alterations may be detected in low-grade disease, inflamed urothelium, post-instrumentation samples, or in the context of synchronous bladder cancer. Therefore, an adverse molecular result should be interpreted as a risk-weighting signal. It may justify repeat ureteroscopic biopsy, selective upper tract sampling, intensified imaging, MDT discussion, or closer surveillance, but it should not override morphologically reassuring findings unless supported by integrated clinical evidence.

A related conceptual barrier is the tendency to conflate diagnostic accuracy, risk enrichment, prognostic association, predictive value, and true decisional utility. A sensitive urine assay that detects UTUC is not automatically a validated KSS-selection tool, and a recurrence marker after RNU is not automatically transferable to surveillance after endoscopic control. The critical question is whether biomarker use improves a predefined decision compared with conventional assessment alone. Logistical problems come in the way of implementation too. The use of selective upper tract urine is not always standardized. Plasma ctDNA assays have different sensitivity with regard to tumor burden. Tumor-guided approaches entail tissue sequencing and analytical turnaround which cannot be consistent across all practices. And since UTUC is a fairly rare disease, it is more difficult to develop sufficiently powered prospective cohorts as it is in the case of bladder cancer. These practical obstacles serve to understand why guidelines are still cautious even in the light of the increasing biomarker literature (8, 9).

This warning is not to be taken as being unsuccessful. It is a natural phase of translation. The biomarker industry is shifting away to discovery detection to clinically ingrained models. However, until such studies are structured with actual KSS decisions, that is, not with generic diagnostic endpoints, the evidence will be suggestive, not decisive.

## 9 Future directions and trial design

The next stage of the field should not simply add new signatures; it should embed the most promising assays into real clinical pathways. Prospective protocols should test biomarkers at decision points where management could change: before choosing between KSS and RNU, when biopsy and imaging are discordant, after KSS when early failure is suspected, after RNU when MRD may influence adjuvant strategies, and during surveillance when repeated endoscopy creates clinical burden. Assay thresholds, specimen source, collection timing, and action rules should be prespecified rather than inferred retrospectively. Endpoint redesign is also necessary. Future studies should not rely only on area under the curve (AUC), sensitivity, or specificity. For KSS, more relevant endpoints include delayed radical surgery, recurrence requiring salvage treatment, renal function preservation, burden of ureteroscopy, treatment-related morbidity, quality of surveillance, and oncologic noninferiority in molecularly selected subgroups. Trials should also prespecify how to adjudicate discordance between ureteroscopic biopsy pathology and liquid-biopsy signals, because using a molecular result to override morphological reassurance requires a higher standard of evidence than current observational studies provide. The third one is the multimodal integration. The probable future does not lie between CTU and biomarker or biopsy and biomarker. It is CTU and cytology and ureteroscopy and pathology and liquid biopsy but not rivalrous (19, 30). Urine-based assays in that integrated model can help decrease diagnostic uncertainty and enhance local triage, whereas plasma ctDNA can be used to enhance biological upstaging and perioperative escalation. The practical objective is not to develop a more complex algorithm. It is in order to minimize the blinds of either modality alone.

Surveillance adaptation deserves particular attention. After KSS, the most valuable trial may not ask whether a biomarker can replace ureteroscopy, but whether molecular results can safely individualize the timing and intensity of monitoring. Such designs must account for temporal discordance: plasma ctDNA clearance or persistence may not align perfectly with local tissue recurrence, and urinary molecular signals may have better local sensitivity but lower spatial specificity when bladder disease is present. This is the type of clinically grounded question that longitudinal liquid biopsy is best suited to test. When these priorities are followed, liquid biopsy would one day raise KSS to a more meaningfully biology-informed strategy rather than a strategy that is predominantly morphology-based. That is the actual vow of the field. It is not the novelty of technology. It is preferable decision-making in the face of uncertainty.

## 10 Conclusions

UTUC is a disease in which treatment decisions are unusually sensitive to imperfections in preoperative risk assessment. KSS has become a central part of modern management, but its safe use depends on selecting tumors that are not only technically manageable but also biologically appropriate for conservative treatment. Conventional work-up remains essential, and current liquid-biopsy evidence does not justify replacing imaging, ureteroscopy, pathology, or MDT judgment. The strongest current position is more limited but clinically meaningful: urine-based assays may improve detection and preoperative triage, whereas plasma and tumor-informed urinary DNA may refine biological upstaging, MRD assessment, and postoperative surveillance. These tools should be interpreted as adjunctive risk-refinement layers, especially in indeterminate-risk cases, and adverse molecular findings should prompt reassessment rather than automatic escalation. The next generation of studies should determine whether biomarker-informed pathways improve treatment allocation, renal preservation, surveillance burden, and oncologic outcomes.

## References

[1] Bray F Laversanne M Sung H Ferlay J Siegel RL Soerjomataram I . Global cancer statistics 2022: GLOBOCAN estimates of incidence and mortality worldwide for 36 cancers in 185 countries. CA A Cancer J Clin. (2024) 74:229–63. doi: 10.3322/caac.21834. PMID: 38572751

[2] Clark CB Matheny M Raman JD . Upper tract urothelial carcinoma: epidemiology, presentation, and high-risk endemic populations. Curr Opin Urol. (2025) 35:53–7. doi: 10.1097/MOU.0000000000001242. PMID: 39465504

[3] Soria F Shariat SF Lerner SP Fritsche H-M Rink M Kassouf W . Epidemiology, diagnosis, preoperative evaluation and prognostic assessment of upper-tract urothelial carcinoma (UTUC). World J Urol. (2017) 35:379–87. doi: 10.1007/s00345-016-1928-x. PMID: 27604375

[4] Petros FG . Epidemiology, clinical presentation, and evaluation of upper-tract urothelial carcinoma. Transl Androl Urol. (2020) 9:1794–8. doi: 10.21037/tau.2019.11.22. PMID: 32944542 PMC7475674

[5] Munoz JJ Ellison LM . Upper tract urothelial neoplasms: incidence and survival during the last 2 decades. J Urol. (2000) 164:1523–5. doi: 10.1016/s0022-5347(05)67019-x 11025695

[6] Raman JD Messer J Sielatycki JA Hollenbeak CS . Incidence and survival of patients with carcinoma of the ureter and renal pelvis in the USA, 1973–2005. BJU Int. (2011) 107:1059–64. doi: 10.1111/j.1464-410X.2010.09675.x. PMID: 20825397

[7] Collà Ruvolo C Nocera L Stolzenbach LF Wenzel M Cucchiara V Tian Z . Incidence and survival rates of contemporary patients with invasive upper tract urothelial carcinoma. Eur Urol Oncol. (2021) 4:792–801. doi: 10.1016/j.euo.2020.11.005. PMID: 33293235

[8] Masson-Lecomte A Birtle A Pradere B Capoun O Compérat E Domínguez-Escrig JL . European association of urology guidelines on upper urinary tract urothelial carcinoma: summary of the 2025 update. Eur Urol. (2025) 87:697–716. doi: 10.1016/j.eururo.2025.02.023. PMID: 40118741

[9] Coleman JA Clark PE Bixler BR Buckley DI Chang SS Chou R . Diagnosis and management of non-metastatic upper tract urothelial carcinoma: AUA/SUO guideline. J Urol. (2023) 209:1071–81. doi: 10.1097/JU.0000000000003480. PMID: 37096584

[10] Pandolfo SD Cilio S Aveta A Wu Z Cerrato C Napolitano L . Upper tract urothelial cancer: Guideline of guidelines. Cancers. (2024) 16:1115. doi: 10.3390/cancers16061115. PMID: 38539450 PMC10969327

[11] Head DJ Raman JD . Kidney-sparing surgery for upper tract urothelial carcinoma—modalities, outcomes, and limitations. JCM. (2024) 13:6593. doi: 10.3390/jcm13216593. PMID: 39518735 PMC11546368

[12] Veccia A Antonelli A Checcucci E Falagario U Carrieri G Guruli G . Segmental ureterectomy for upper tract urothelial carcinoma: A systematic review and meta-analysis of comparative studies. Clin Genitourin Cancer. (2020) 18:e10–20. doi: 10.1016/j.clgc.2019.10.015. PMID: 31704265

[13] Kleinmann N Matin SF Pierorazio PM Gore JL Shabsigh A Hu B . Primary chemoablation of low-grade upper tract urothelial carcinoma using UGN-101, a mitomycin-containing reverse thermal gel (OLYMPUS): an open-label, single-arm, phase 3 trial. Lancet Oncol. (2020) 21:776–85. doi: 10.1016/S1470-2045(20)30147-9. PMID: 32631491

[14] Yip W Sjoberg DD Nogueira LM Tracey AT Alvim RG Reisz PA . Final results of a phase I trial of WST-11 (TOOKAD Soluble) vascular-targeted photodynamic therapy for upper tract urothelial carcinoma. J Urol. (2023) 209:863–71. doi: 10.1097/JU.0000000000003202. PMID: 36724067 PMC10265489

[15] Lasmanovich R Shvero A Kleinmann N . Upper tract urothelial carcinoma: conservative management - intraluminal adjuvant therapy, and surveillance. Curr Opin Urol. (2025) 35:68–74. doi: 10.1097/MOU.0000000000001240. PMID: 39483069

[16] Levy A Canes D . Perioperative complications and adverse sequelae of radical nephroureterectomy. Transl Androl Urol. (2020) 9:1853–9. doi: 10.21037/tau.2019.12.25. PMID: 32944549 PMC7475668

[17] Padullés B Carrasco R Ingelmo-Torres M Roldán FL Gómez A Vélez E . Prognostic value of liquid-biopsy-based biomarkers in upper tract urothelial carcinoma. IJMS. (2024) 25:3695. doi: 10.3390/ijms25073695. PMID: 38612507 PMC11012136

[18] Xiao H Yi L Ma Z Dai K Liu Y Gao Y . Urine-based biomarkers in the diagnosis of upper tract urothelial carcinoma: A systematic review and meta-analysis. JCM. (2026) 15:1612. doi: 10.3390/jcm15041612. PMID: 41753299 PMC12941476

[19] Rose KM Huelster HL Meeks JJ Faltas BM Sonpavde GP Lerner SP . Circulating and urinary tumour DNA in urothelial carcinoma — upper tract, lower tract and metastatic disease. Nat Rev Urol. (2023) 20:406–19. doi: 10.1038/s41585-023-00725-2. PMID: 36977797

[20] Subiela JD Territo A Mercadé A Balañà J Aumatell J Calderon J . Diagnostic accuracy of ureteroscopic biopsy in predicting stage and grade at final pathology in upper tract urothelial carcinoma: Systematic review and meta-analysis. Eur J Surg Oncol. (2020) 46:1989–97. doi: 10.1016/j.ejso.2020.06.024. PMID: 32674841

[21] Margolin EJ Matulay JT Li G Meng X Chao B Vijay V . Discordance between ureteroscopic biopsy and final pathology for upper tract urothelial carcinoma. J Urol. (2018) 199:1440–5. doi: 10.1016/j.juro.2018.02.002. PMID: 29427584

[22] Zhang H Xu Y Wang K Zheng C Li Y Gong H . Large-scale prospective validation study of a multiplex RNA urine test for noninvasive detection of upper tract urothelial carcinoma. Eur Urol Oncol. (2024) 7:1384–93. doi: 10.1016/j.euo.2024.03.005. PMID: 38523018

[23] Nakano K Koh Y Yamamichi G Yumiba S Tomiyama E Matsushita M . Perioperative circulating tumor DNA enables the identification of patients with poor prognosis in upper tract urothelial carcinoma. Cancer Sci. (2022) 113:1830–42. doi: 10.1111/cas.15334. PMID: 35293110 PMC9128184

[24] Huelster HL Gould B Schiftan EA Camperlengo L Davaro F Rose KM . Novel use of circulating tumor DNA to identify muscle-invasive and non–organ-confined upper tract urothelial carcinoma. Eur Urol. (2024) 85:283–92. doi: 10.1016/j.eururo.2023.09.017. PMID: 37802683

[25] Tamura D Abe M Hiraki H Sasaki N Yashima‐Abo A Ikarashi D . Postoperative recurrence detection using individualized circulating tumor DNA in upper tract urothelial carcinoma. Cancer Sci. (2024) 115:529–39. doi: 10.1111/cas.16025. PMID: 38083992 PMC10859621

[26] Powles T Assaf ZJ Davarpanah N Banchereau R Szabados BE Yuen KC . ctDNA guiding adjuvant immunotherapy in urothelial carcinoma. Nature. (2021) 595:432–7. doi: 10.1038/s41586-021-03642-9. PMID: 34135506

[27] Stewart TF Chalfin H Simon N Tan A Apolo A McKay RR . Perioperative use of ctDNA to guide treatment for urothelial carcinoma: The future is now. Bladder Cancer. (2024) 10:183–98. 39493820 10.3233/BLC-230105 PMC11530029

[28] Kato T Yajima S Nakamura Y Kobayashi S Miyake H . Current status and future perspectives of molecular residual disease testing in genitourinary cancers. Int J Urol. (2025) 32:1735–45. doi: 10.1111/iju.70215. PMID: 40955810 PMC12687926

[29] Rouprêt M Seisen T Birtle AJ Capoun O Compérat EM Dominguez-Escrig JL . European association of urology guidelines on upper urinary tract urothelial carcinoma: 2023 update. Eur Urol. (2023) 84:49–64. doi: 10.1016/j.eururo.2023.03.013. PMID: 36967359

[30] Zganjar AJ Thiel DD Lyon TD . Diagnosis, workup, and risk stratification of upper tract urothelial carcinoma. Transl Androl Urol. (2023) 12:1456–68. doi: 10.21037/tau-23-45. PMID: 37814699 PMC10560346

[31] Schuil HW Figaroa OJA Baard J Lifshitz DA Jamaludin FS Kamphuis GM . Comparison of surgical effectiveness: kidney sparing surgery for upper tract urothelial carcinoma. Curr Opin Urol. (2025) 35:58–67. doi: 10.1097/MOU.0000000000001248. PMID: 39512154

[32] Ko ICH Wong CHM Leung DKW Liu AQ Cheng KCK Siu BWH . Kidney-sparing approach for upper tract urothelial carcinoma: An update on current evidence. Asian J Urol. (2025) 12:295–308. doi: 10.1016/j.ajur.2024.08.003. PMID: 41049824 PMC12490676

[33] Seisen T Peyronnet B Dominguez-Escrig JL Bruins HM Yuan CY Babjuk M . Oncologic outcomes of kidney-sparing surgery versus radical nephroureterectomy for upper tract urothelial carcinoma: A systematic review by the EAU Non-muscle Invasive Bladder Cancer Guidelines Panel. Eur Urol. (2016) 70:1052–68. doi: 10.1016/j.eururo.2016.07.014. PMID: 27477528

[34] Matin SF Pierorazio PM Kleinmann N Gore JL Shabsigh A Hu B . Durability of response to primary chemoablation of low-grade upper tract urothelial carcinoma using UGN-101, a mitomycin-containing reverse thermal gel: OLYMPUS trial final report. J Urol. (2022) 207:779–88. doi: 10.1097/JU.0000000000002350. PMID: 34915741 PMC12721675

[35] Foerster B D’Andrea D Abufaraj M Broenimann S Karakiewicz PI Rouprêt M . Endocavitary treatment for upper tract urothelial carcinoma: A meta-analysis of the current literature. Urologic Oncol Semin Original Investigations. (2019) 37:430–6. doi: 10.1016/j.urolonc.2019.02.004. PMID: 30846387

[36] Xylinas E Colin P Audenet F Phe V Cormier L Cussenot O . Intravesical recurrence after radical nephroureterectomy for upper tract urothelial carcinomas: predictors and impact on subsequent oncological outcomes from a national multicenter study. World J Urol. (2013) 31:61–8. doi: 10.1007/s00345-012-0957-3. PMID: 23053211

[37] Katims AB Say R Derweesh I Uzzo R Minervini A Wu Z . Risk factors for intravesical recurrence after minimally invasive nephroureterectomy for upper tract urothelial cancer (ROBUUST Collaboration). J Urol. (2021) 206:568–76. doi: 10.1097/JU.0000000000001786. PMID: 33881931

[38] Potretzke AM Knight BA Vetter JM Anderson BG Hardi AC Bhayani SB . Diagnostic utility of selective upper tract urinary cytology: A systematic review and meta-analysis of the literature. Urology. (2016) 96:35–43. doi: 10.1016/j.urology.2016.04.030. PMID: 27151340

[39] Zhang ML VandenBussche CJ Hang J-F Miki Y McIntire PJ Peyton S . A review of urinary cytology in the setting of upper tract urothelial carcinoma. J Am Soc Cytopathology. (2021) 10:29–35. doi: 10.1016/j.jasc.2020.06.011. PMID: 32792229

[40] Zhang ML Miki Y Hang J Vohra M Peyton S McIntire PJ . A review of upper urinary tract cytology performance before and after the implementation of The Paris System. Cancer Cytopathol. (2021) 129:264–74. doi: 10.1002/cncy.22343. PMID: 32897658

[41] Gravestock P Cullum D Somani B Veeratterapillay R . Diagnosing upper tract urothelial carcinoma: A review of the role of diagnostic ureteroscopy and novel developments over last two decades. Asian J Urol. (2024) 11:242–52. doi: 10.1016/j.ajur.2022.08.003. PMID: 38680592 PMC11053284

[42] Territo A Gallioli A Meneghetti I Fontana M Huguet J Palou J . Diagnostic ureteroscopy for upper tract urothelial carcinoma: friend or foe? Arab J Urol. (2021) 19:46–58. doi: 10.1080/2090598X.2021.1883810. PMID: 33763248 PMC7954478

[43] Simon CT Skala SL Weizer AZ Ambani SN Chinnaiyan AM Palapattu G . Clinical utility and concordance of upper urinary tract cytology and biopsy in predicting clinicopathological features of upper urinary tract urothelial carcinoma. Hum Pathol. (2019) 86:76–84. doi: 10.1016/j.humpath.2018.11.021. PMID: 30537495

[44] Jue JS Alameddine M Armenakas NA . Overcoming understaging and undergrading in upper tract urothelial carcinoma. Comment on Ghoreifi et al. Modern kidney-sparing management of upper tract urothelial carcinoma. Cancers 2023, 15, 4495. Cancers. (2024) 16:1002. doi: 10.3390/cancers16051002. PMID: 38473362 PMC10931491

[45] Jaime-Casas S Tripathi A Pal SK Yip W . Clinical implications of the molecular and genomic landscape of upper tract urothelial carcinoma. Curr Urol Rep. (2025) 26:11. doi: 10.1007/s11934-024-01245-1. PMID: 39379745 PMC11461588

[46] Sfakianos JP Cha EK Iyer G Scott SN Zabor EC Shah RH . Genomic characterization of upper tract urothelial carcinoma. Eur Urol. (2015) 68:970–7. doi: 10.1016/j.eururo.2015.07.039. PMID: 26278805 PMC4675454

[47] Fujii Y Sato Y Suzuki H Kakiuchi N Yoshizato T Lenis AT . Molecular classification and diagnostics of upper urinary tract urothelial carcinoma. Cancer Cell. (2021) 39:793–809.e8. doi: 10.1016/j.ccell.2021.05.008. PMID: 34129823 PMC9110171

[48] Sfakianos JP Gul Z Shariat SF Matin SF Daneshmand S Plimack E . Genetic differences between bladder and upper urinary tract carcinoma: Implications for therapy. Eur Urol Oncol. (2021) 4:170–9. doi: 10.1016/j.euo.2020.12.007. PMID: 33386276

[49] Ghoreifi A Ladi-Seyedian S-S Piatti P Chew YC Jara B Sanossian L . A urine-based DNA methylation marker test to detect upper tract urothelial carcinoma: A prospective cohort study. J Urol. (2023) 209:854–62. doi: 10.1097/JU.0000000000003188. PMID: 36795966

[50] Yang G Zeng S He W Zhang L Xu C Pan J . Clinical validation of UroCAD test for upper tract urothelial carcinoma detection: results from a prospective multi-center study. J Hematol Oncol. (2026) 19:8. doi: 10.1186/s13045-025-01772-5. PMID: 41508041 PMC12784582

[51] Freund JE Liem EIML Savci-Heijink CD De Reijke TM . Fluorescence in situ hybridization in 1 mL of selective urine for the detection of upper tract urothelial carcinoma: a feasibility study. Med Oncol. (2019) 36:10. doi: 10.1007/s12032-018-1237-x. PMID: 30499061 PMC6267383

[52] Su X Hao H Li X He Z Gong K Zhang C . Fluorescence in situ hybridization status of voided urine predicts invasive and high-grade upper tract urothelial carcinoma. Oncotarget. (2017) 8:26106–11. doi: 10.18632/oncotarget.15344. PMID: 28212539 PMC5432242

[53] Sassa N Iwata H Kato M Murase Y Seko S Nishikimi T . Diagnostic utility of UroVysion combined with conventional urinary cytology for urothelial carcinoma of the upper urinary tract. Am J Clin Pathol. (2019) 151:469–78. doi: 10.1093/ajcp/aqy170. PMID: 30668617

[54] Białek Ł Bilski K Dobruch J Krajewski W Szydełko T Kryst P . Non-invasive biomarkers in the diagnosis of upper urinary tract urothelial carcinoma—a systematic review. Cancers. (2022) 14:1520. doi: 10.3390/cancers14061520. PMID: 35326672 PMC8945959

[55] Territo A Gallioli A Diana P Boissier R Fontana M Gaya JM . DNA methylation urine biomarkers test in the diagnosis of upper tract urothelial carcinoma: results from a single-center prospective clinical trial. J Urol. (2022) 208:570–9. doi: 10.1097/JU.0000000000002748. PMID: 35549312

[56] Ye J Wang X Liao X Chen Y Chen Z Wang Q . DNA methylation urine test in the diagnosis of upper tract urothelial carcinoma: a systematic review and meta-analysis. Int J Surg. (2025) 111:1255–64. doi: 10.1097/JS9.0000000000001904. PMID: 39037716 PMC11745744

[57] Palermo M D’Elia C Trenti E Comploj E Mian C Schwienbacher C . Prospective evaluation of the RT-PCR based urinary marker Bladder Epicheck® as a diagnostic tool in upper urinary tract tumor. Minerva Urol Nephrol. (2024) 76:195–202. doi: 10.23736/S2724-6051.23.05488-5. PMID: 38498297

[58] Wei W Fan P Zhang Z Wu D Liu J Wang L . A urine-based liquid biopsy for detection of upper tract urothelial carcinoma: a self-matched study. BMC Cancer. (2024) 24:1180. doi: 10.1186/s12885-024-12913-3. PMID: 39333973 PMC11438001

[59] Lin Y Lin L Yang Y Li M Jiang X Fu T . DNA methylation architecture provides insight into the pathogenesis of upper tract urothelial carcinoma: a systematic review and meta-analysis. Clin Genitourin Cancer. (2023) 21:32–42. doi: 10.1016/j.clgc.2022.10.008. PMID: 36376170

[60] Hayashi Y Fujita K Matsuzaki K Matsushita M Kawamura N Koh Y . Diagnostic potential of TERT promoter and FGFR 3 mutations in urinary cell‐free DNA in upper tract urothelial carcinoma. Cancer Sci. (2019) 110:1771–9. doi: 10.1111/cas.14000. PMID: 30887605 PMC6501003

[61] Xu Y Ma X Ai X Gao J Liang Y Zhang Q . A urine-based liquid biopsy method for detection of upper tract urinary carcinoma. Front Oncol. (2021) 10:597486. doi: 10.3389/fonc.2020.597486. PMID: 33634022 PMC7901537

[62] Ouyang W Luo L Zhang J Xu R Lu Q Xu Z . Urine cellular DNA point mutation and methylation for identifying upper tract urinary carcinoma. Cancers. (2022) 14:3537. doi: 10.3390/cancers14143537. PMID: 35884598 PMC9319988

[63] Fujita K Miyake M Hashimoto M Shimizu T Linehan J Gottlieb J . Diagnostic accuracy of the Oncuria-Detect multiplex immunoassay in detecting upper tract urothelial carcinoma. J Urol. (2026) 215:174–82. doi: 10.1097/JU.0000000000004803. PMID: 41042706 PMC12794688

[64] Crocetto F Amicuzi U Musone M Magliocchetti M Di Lieto D Tammaro S . Liquid biopsy: current advancements in clinical practice for bladder cancer. J Liquid Biopsy. (2025) 9:100310. doi: 10.1016/j.jlb.2025.100310. PMID: 40698358 PMC12281373

[65] Birtle AJ Jones R Chester J Lewis R Biscombe K Johnson M . Improved disease-free survival with adjuvant chemotherapy after nephroureterectomy for upper tract urothelial cancer: final results of the POUT trial. JCO. (2024) 42:1466–71. doi: 10.1200/JCO.23.01659. PMID: 38350047 PMC11095877

[66] Powles T Assaf ZJ Degaonkar V Grivas P Hussain M Oudard S . Updated overall survival by circulating tumor DNA status from the phase 3 IMvigor010 trial: adjuvant atezolizumab versus observation in muscle-invasive urothelial carcinoma. Eur Urol. (2024) 85:114–22. doi: 10.1016/j.eururo.2023.06.007. PMID: 37500339

[67] Bellmunt J Russell BM Szabados B Valderrama BP Nadal R . Current and future role of circulating DNA in the diagnosis and management of urothelial carcinoma. Am Soc Clin Oncol Educ Book. (2025) 45:e471912. doi: 10.1200/EDBK-25-471912. PMID: 39883890

[68] Lonati C Moschini M Simeone C Spiess PE Necchi A . Lynch syndrome in urological practice: diagnosis, therapeutic strategies, and screening for upper tract urothelial carcinoma. Curr Opin Urol. (2022) 32:40–7. doi: 10.1097/MOU.0000000000000936. PMID: 34608026

[69] Rasmussen M Madsen MG Therkildsen C . Immunohistochemical screening of upper tract urothelial carcinomas for Lynch syndrome diagnostics: a systematic review. Urology. (2022) 165:44–53. doi: 10.1016/j.urology.2022.02.006. PMID: 35217028

[70] Wu J Jin S Gu C Wei Y Zhu Y Necchi A . Inherited mutations in Chinese patients with upper tract urothelial carcinoma. Cell Rep Med. (2023) 4:100883. doi: 10.1016/j.xcrm.2022.100883. PMID: 36630951 PMC9873949

[71] Bicocca VT Phillips KG Fischer DS Caruso VM Goudarzi M Garcia-Ransom M . Urinary comprehensive genomic profiling correlates urothelial carcinoma mutations with clinical risk and efficacy of intervention. J Clin Med. (2022) 11:5827. doi: 10.3390/jcm11195827. PMID: 36233691 PMC9571552

[72] Pepe L Fiorentino V Pizzimenti C Riganati G FranChina M Micali M . The simultaneous use of Bladder EpiCheck and urinary cytology can improve the sensitivity and specificity of diagnostic follow-up of urothelial lesions: up-to-date data from a multi-institutional cohort. Diseases. (2024) 12:219. doi: 10.3390/diseases12090219. PMID: 39329888 PMC11431392

[73] Pierconti F Rossi ED Cenci T Carlino A Fiorentino V Totaro A . DNA methylation analysis in urinary samples: a useful method to predict the risk of neoplastic recurrence in patients with urothelial carcinoma of the bladder in the high-risk group. Cancer Cytopathol. (2023) 131:158–64. doi: 10.1002/cncy.22657. PMID: 36262084

[74] Germana E Pepe L Pizzimenti C Ballato M Pierconti F Tuccari G . Programmed cell death ligand 1 (PD-L1) immunohistochemical expression in advanced urothelial bladder carcinoma: an updated review with clinical and pathological implications. Int J Mol Sci. (2024) 25:6750. doi: 10.3390/ijms25126750. PMID: 38928456 PMC11203574

[75] Hurel S Roupret M Seisen T Comperat E Phe V Droupy S . Influence of preoperative factors on the oncologic outcome for upper urinary tract urothelial carcinoma after radical nephroureterectomy. World J Urol. (2015) 33:335–41. doi: 10.1007/s00345-014-1311-8. PMID: 24810657

